# Different Patterns of Hearing Loss among Tinnitus Patients: A Latent Class Analysis of a Large Sample

**DOI:** 10.3389/fneur.2017.00046

**Published:** 2017-02-20

**Authors:** Berthold Langguth, Michael Landgrebe, Winfried Schlee, Martin Schecklmann, Veronika Vielsmeier, Thomas Steffens, Susanne Staudinger, Hannah Frick, Ulrich Frick

**Affiliations:** ^1^Department of Psychiatry and Psychotherapy, University of Regensburg, Regensburg, Germany; ^2^Interdisciplinary Tinnitus Center of the University of Regensburg, Regensburg, Germany; ^3^kbo Lech-Mangfall-Klinik Agatharied, Hausham, Germany; ^4^Department of Otorhinolaryngology, University of Regensburg, Regensburg, Germany; ^5^Department of Statistical Science, University College London, London, UK; ^6^HSD University of Applied Sciences, Cologne, Germany

**Keywords:** chronic tinnitus, hearing loss, cluster analysis, latent classes, audiometry

## Abstract

**Background:**

The heterogeneity of tinnitus is a major challenge for tinnitus research. Even if a complex interaction of many factors is involved in the etiology of tinnitus, hearing loss (HL) has been identified as the most relevant etiologic factor. Here, we used a data-driven approach to identify patterns of hearing function in a large sample of tinnitus patients presenting in a tinnitus clinic.

**Methods:**

Data from 2,838 patients presenting at the Tinnitus Center of the University Regensburg between 2007 and 2014 have been analyzed. Standard audiometric data were frequency-wise categorized in four categories [a: normal hearing (0–20 dB HL); b: moderate HL (25–50 dB HL; representing outer hair cell loss); c: severe HL (>50 dB HL; representing outer and inner hair cell loss); d: no data available] and entered in a latent class analysis, a statistical method to find subtypes of cases in multivariate categorical data. To validate the clinical relevance of the identified latent classes, they were compared with respect to clinical and demographic characteristics of their members.

**Results:**

The classification algorithm identified eight distinct latent classes with an excellent separation. Patient classes differed with respect to demographic (e.g., age, gender) and clinical characteristics (e.g., tinnitus location, tinnitus severity, gradual, or abrupt onset, etc.).

**Discussion:**

Our results demonstrate that data-driven categorization of hearing function seems to be a promising approach for profiling tinnitus patients, as it revealed distinct subtypes that reflect prototypic forms of HL and that differ in several relevant clinical characteristics.

## Introduction

Tinnitus, the perception of sound in the absence of a corresponding auditory stimulus, is a frequent disorder (1). Clinically, tinnitus can be very heterogeneous with respect to the perceived sound characteristics (e.g., tonal vs. broadband noise), its localization (in one or both ears, in the head, etc.), its time course (continuous, intermittent, fluctuating), its modifying factors (e.g., reduction by masking), and its comorbidities (hyperacusis, depression, insomnia). This clinical heterogeneity is paralleled by heterogeneity in tinnitus pathophysiology. Recent pathophysiological models assume that tinnitus emerges as a clinical symptom as result of abnormal activation of different overlapping and interacting brain networks (2). Abnormally activated networks in tinnitus patients include the auditory, attention, salience, and distress networks. The activation pattern varies from patient to patient and reflects the individuals’ symptoms (3, 4). As an example, distressed and not distressed tinnitus patients differ in their activation of the cortical stress-related network (5).

Among other factors, the large heterogeneity of the tinnitus patient population represents a major barrier for the development of effective tinnitus treatments [see, e.g., Ref. (4) for a review]. The heterogeneity of tinnitus can be described on various dimensions such as its etiology, perceptual characteristics of the sound (i.e., pitch and loudness), time since onset, continuous or intermittent, levels of conscious awareness and perceived distress, and comorbidities (6). One approach to address this challenge is the establishment of large databases for enabling data-driven identification of subtypes (7–9).

Even if current etiologic models assume a complex interplay of various factors, several lines of evidence indicate that hearing loss (HL) is the most relevant etiologic factor for tinnitus development (10, 11). First, epidemiological studies have identified HL as a major risk factor for tinnitus (12). Second, induction of HL in animals induces reliably increased neuronal activity and synchronicity (11, 13) as well as behavioral evidence of tinnitus (14). Third, the tinnitus spectrum of most tinnitus patients is clearly related to their pattern of HL (15, 16). If, for example, somebody experiences tinnitus at 4 kHz at the left ear, typically a HL at 4 kHz on the left ear can be detected in the audiogram.

Because of the high etiological relevance of HL for tinnitus, hearing function is presumably one of the relevant criteria for classifying tinnitus patients. Currently, this factor is not receiving major attention in the classification of tinnitus patients. In the description of study samples in tinnitus research rarely details are given about the hearing function of participants. If anything, the mean audiogram (averaged over all participants) is displayed. Whenever statistical analyses are performed relating hearing function to other aspects of tinnitus, typically either HL averaged over both ears and all measured frequencies or the maximum HL is used to characterize patients’ incapacities.

Both indicators are of only limited value, as there exist different patterns of the quantity of HL, e.g., related to outer or inner hair cell damage, which might be highly relevant for a comprehensive characterization of a tinnitus patient. However, the information about specific patterns of HL quantity is getting lost, if the audiograms of participants are averaged.

Here, we used a data-driven approach to identify patterns of hearing function in a large sample of tinnitus patients presenting in a specialized Tinnitus Center. The goal of the study was to clarify, whether specific patterns of HL quantity can be established from patients’ audiograms and how stable different tinnitus patients can be classified using these patterns of HL. In case, this data-driven approach for pattern identification reveals a statistically sound and medically well interpretable solution, one could assume that this new categorization of hearing function would be a reasonable alternative to current approaches to deal with audiogram data in the characterization of tinnitus patients.

## Materials and Methods

### Patient Assessment

The analysis has been based on data from all patients who presented between 2007 and 2014 at the Interdisciplinary Tinnitus Center at the University of Regensburg, Regensburg, Germany and who gave informed consent for data collection in the Tinnitus Research Initiative database (TRI Database). The TRI Database is an international patient database that has been established for facilitating research efforts toward the identification of tinnitus subtypes and outcome predictors (8). For these purposes, patients presenting in tinnitus clinics and undergoing specific, well-defined treatment interventions, both in clinical trials or in clinical routine, are systematically assessed and their data are pre-processed for plausibility in standardized protocols (8). In addition to audiometric data, the database includes demographic and clinical data, data about tinnitus handicap, tinnitus severity, and quality of life [for a detailed description of the datasets, see Ref. (8)]. Collection of data for the TRI Database has been approved by the local ethics committee of the University of Regensburg, Germany. All patients completed various tinnitus questionnaires, underwent a microscopy of the ear and received an audiological examination including pure-tone audiometry (125–8,000 Hz). Sample size for this classificatory part of our analysis thus reached *n* = 2,838.

Due to item-wise missing values of the 17 variables used to describe demographic or clinical characteristics of patients beyond the results of the audiometry, effective sample sizes in comparisons of emerging latent classes (see below) vary. Most variables reach effective sample sizes clearly above 2,000 patients. Only Beck Depression Inventory (BDI) (*n* = 1,544) and TBF-total score (*n* = 1,464), which both were added later to the assessment program, and patients’ CGI-ratings (*n* = 807), which were assessed only in patients entering a treatment program at their first day of treatment, reached smaller sample sizes.

### Classification of Hearing Function

As HL presenting at different threshold levels can be linked to different possible pathological mechanisms (17), patients’ audiogram data were not treated as a “naive” continuous metric of intensity of HL, but classified into four different states. At each of seven pre-specified frequencies (125 Hz, 250 Hz, 500 Hz, 1 kHz, 2 kHz, 4 kHz, and 8 kHz) the measured grade of HL was classified into one of the following four categories: (1) normal hearing (0–20 dB HL); (2) mild/moderate HL (25–50 dB HL), representing mostly outer hair cell loss; (3) severe/profound HL (>50 dB HL), representing outer and inner hair cell damage; and (4) no data available (either due to technical restrictions or because of physician based abbreviations of the audiogram assessment). This categorization tries to better reflect the physiological condition of patients’ hearing and to avoid a potentially misleading interpretation of HL as homogenously quantifiable risk factor (18). Additionally, by introducing a fourth category of “not measured” into the analysis, a potential selection bias due to technical equipment or due to physicians’ practices in assessing HL can be avoided.

### Statistical Methods

Latent class analysis (LCA) is a statistical approach for identifying groups of cases in multivariate categorical data. These groups are called latent classes. Our assumption is that HL of the left ear does not predetermine HL for the right side, and *vice versa*. Therefore, a vector of 14 variables (each frequency with 4 HL categories) per patient defines the starting point of statistical analysis. Theoretically, over 250 million combinations (exactly 4**14 = 268,435,456) are possible. Observed were 590 answering vectors in 2,838 patients. LCA tries to reproduce the empirical frequencies of these answering vectors by estimating two different kinds of model parameters: π*_g_*, i.e., the relative sizes of *G* latent classes (*G* has to be determined *a priori*), and the probability π*_isg_* for an answer *s* (*s* = 1, …, 4) on each item *i* (*i* = 1, …, 14), given the membership in a certain latent class *g*. The class-specific answering probabilities π*_ix_*_|_*_g_* thus indicate the nearness between this specific answer and membership in the respective latent class *g*. LCA therefore results in *G* membership probabilities per person to each of the latent classes (see Supplementary Material for further details). Strong solutions with little overlap between different latent profiles provide for each person one unequivocal high membership probability and *m* − 1 very low membership probabilities. Classification then is based on the modal value of these probabilities.

Visualization of membership probabilities is an intuitively appealing method of model evaluation. Alternatively, so-called fit indices can be calculated for each number of latent classes chosen. Clearly, a perfect model fit must be reached, if (in our case) 590 classes are introduced to the model. By introducing a penalty term for adding new latent classes, a decision for the optimal number of classes can be drawn choosing the model with the best fit. We used the BIC index as criteria to decide on the number of latent classes. Calculations were performed using WinMIRA by von Davier (19).

Differences between latent classes on continuous variables (like age) were assessed using SAS PROC GLM to perform analysis of variance for unequal cell sizes. Differences on qualitative variables (like sex) were assessed using chi-square test (SAS PROC FREQ). Due to the exploratory character of this study, no adjustment for type-I error inflation was performed.

## Results

The sample comprised 2,838 patients (mean age 51.7 ± 12.9 years, 67.6% male). In 1,925 of them, audiometric data were available.

In order to avoid local maxima of the estimation function, 50 starting values for parameter estimation were randomly chosen for each model covering 2 up to 12 latent classes. According to the BIC fit index, eight latent classes represent an optimal solution for the given data set. Posterior probabilities of class membership display excellent separation of groups of HL as indicated by a mean membership probability above 0.9 for all latent classes (Table 1) (see Supplementary Material for details about the calculation of latent classes). Detailed clinical and demographic data of the sample are given in Table 2.

**Table 1:** **Mean membership probabilities for latent classes**.

Patient is classified into		Mean membership probabilities for latent classes							
Class no.	Relative class size	LC1	LC2	LC3	LC4	LC5	LC6	LC7	LC8
LC1	0.322	1.000	0.000	0.000	0.000	0.000	0.000	0.000	0.000
LC2	0.216	0.000	0.910	0.058	0.030	0.000	0.001	0.000	0.000
LC3	0.206	0.000	0.054	0.943	0.003	0.000	0.001	0.000	0.000
LC4	0.133	0.000	0.057	0.004	0.914	0.009	0.012	0.004	0.000
LC5	0.048	0.000	0.000	0.000	0.018	0.967	0.009	0.003	0.004
LC6	0.037	0.000	0.002	0.000	0.019	0.012	0.963	0.004	0.000
LC7	0.029	0.000	0.006	0.000	0.009	0.013	0.004	0.968	0.000
LC8	0.011	0.000	0.000	0.000	0.000	0.002	0.000	0.000	0.998

**Table 2:** **Patterns of HL and related demographic and clinical data**.

Description of patterns of HL											
Variable	Pattern of hearing loss (HL)	LC2: bilateral high frequency (HF) HL	LC3: normal hearing	LC4: bilateral medium-HF HL	LC5: severe pantonal HL	LC6: left-sided pantonal medium HL	LC7: right-sided pantonal severe HL	LC8: left-sided pantonal severe HL	LC1: lacking audiometry	Total	Prob. *F*-test
	*N*^a^	614	581	378	135	104	81	32	913	2.838	
Prevalence (adjusted)	(without LC1)	0.319	0.302	0.196	0.070	0.054	0.042	0.017	n.a.	1.00	n.a.

Age	Mean	53.099	41.878	58.706	61.186	53.799	57.026	57.372	51.763	51.694	*p* < 0.0001
	SD	9.76	11.32	10.94	12.78	10.92	12.51	12.69	13.00	12.94	

Age at onset	Mean	45.201	41.726	47.869	47.944	45.765	46.908	44.630	43.053	43.213	*p* < 0.0001
	SD	11.92	11.98	13.51	16.61	10.92	14.66	16.91	14.08	13.85	

Tinnitus Questionnaire total score (at screening)	Mean	40.178	36.813	41.894	50.944	44.768	42.519	46.500	41.639	40.910	*p* < 0.0001
	SD	17.35	17.19	16.83	17.09	17.35	17.95	17.88	18.31	17.76	

Beck Depression Inventory total score (at screening)	Mean	10.117	10.835	10.086	14.356	10.970	11.188	11.409	11.532	10.905	*p* < 0.01
	SD	8.04	8.62	7.70	9.84	10.11	7.95	8.74	9.05	8.60	

TBF-12 total score (at screening)	Mean	12.772	11.970	13.158	14.971	13.563	13.083	14.733	12.706	12.801	*p* < 0.01
	SD	5.10	5.58	5.28	4.90	0.96	5.76	5.20	5.68	5.42	

Severity rating (patient, at screening)	Mean	3.407	3.219	3.548	3.813	3.437	3.472	3.567	3.486	3.431	*p* < 0.0001
	SD	0.84	0.90	0.81	0.83	0.84	0.87	0.82	0.90	0.88	

CGI rating (patient, at first visit)	Mean	3.880	3.978	3.769	3.800	4.094	3.920	4.133	3.909	3.901	n.s.
	SD	0.79	0.78	0.80	0.94	0.96	0.91	0.92	0.80	0.82	

**Categorical variables**											**Prob. chi-square test**

Sex	% Female	24.9	36.7	27.0	50.4	50.0	46.9	46.9	30.6	32.4	*p* < 0.001

Handedness	% Right hand	83.3	84.1	83.1	87.9	80.6	73.8	78.1	83.8	83.3	n.s.

Abrupt beginning of tinnitus	% Abrupt	48.8	56.8	43.1	40.0	60.4	60.3	57.1	52.3	51.1	*p* < 0.001

Tinnitus pulsation	% No	82.2	80.0	82.3	72.7	81.5	66.2	75.0	77.9	79.6	*p* < 0.01

Location of tinnitus^b^	% Right	8.6	14.3	10.0	9.9	4.0	48.2	6.3	12.9	12.3	*p* < 0.001
	% Left	18.2	13.1	15.2	15.2	37.6	11.1	37.5	17.7	17.1	
	% Both sides (worse left)	18.2	22.6	26.3	21.2	21.8	6.2	34.4	18.7	20.6	
	% Both sides (worse right)	16.7	16.6	17.6	21.2	12.9	23.5	6.3	17.2	17.1	
	% Both sides equally	27.8	24.5	20.6	24.2	13.9	8.6	6.3	23.8	23.4	

Tinnitus manifestation over time	% Constant	87.9	82.8	88.8	92.0	88.5	80.8	93.3	84.4	86.1	*p* < 0.05

Loudness variation	% Yes	60.4	61.1	55.5	64.0	63.8	59.2	63.3	61.5	60.4	n.s.

Tonal characteristic of tinnitus^c^	% Tone	64.8	65.6	58.3	45.2	50.5	53.3	33.3	63.7	61.3	*p* < 0.001
	% Noise	5.7	10.6	11.0	32.5	19.0	23.4	33.3	11.3	11.9	
	% Crickets	21.5	15.6	20.7	12.7	15.8	13.0	16.7	16.1	17.7	

Tinnitus pitch	% Very high	30.8	27.8	23.6	27.4	28.1	18.7	30.0	29.1	27.9	*p* < 0.001
	% High	56.1	51.8	55.8	53.0	42.2	41.3	46.7	52.0	52.8	
	% Medium	11.9	16.0	19.4	16.2	22.9	40.0	20.0	16.6	16.8	
	% Low	1.3	4.4	1.2	3.4	6,3	0.0	3.3	2.3	2.5	

*^a^Due to missing values, sample sizes might vary*.

^b^Lacking categories to 100% are “inside head,” “elsewhere.”

^c^Lacking category is “other.”

**Figure 1:**
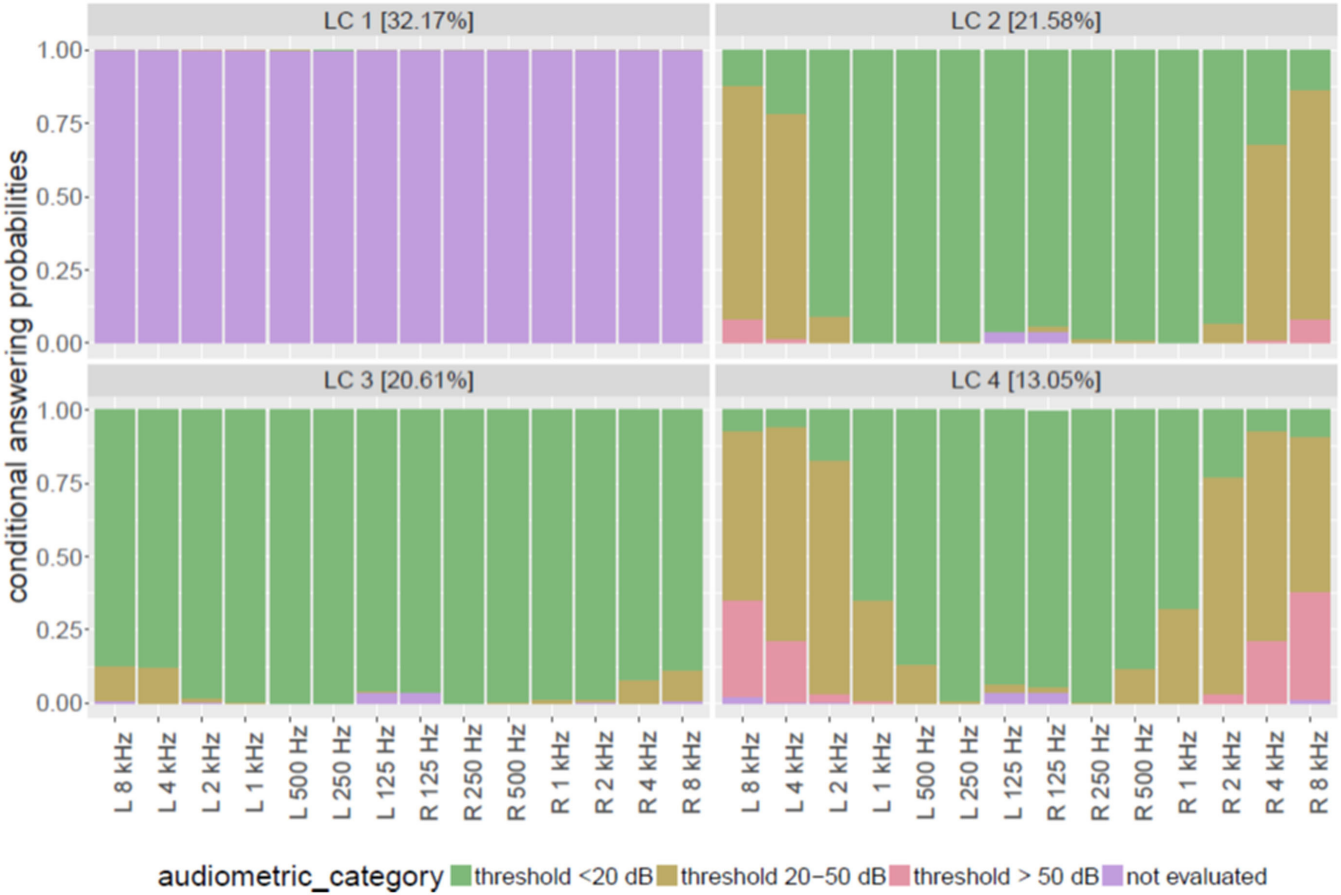
**Patterns of hearing loss with high prevalence in tinnitus patients**.

The largest class (LC1; Figure 1 upper left chart) comprises nearly one-third (32.2%) of the sample and represents patients with lacking audiometry. By holding these untested patients in a separate group it is possible to scrutinize potential selection biases between clinical characteristics and audiometry. Therefore, it is meaningful to analyze these patients as a specific “pattern of hearing loss.”

The 21.6% of the sample suffers from mild to moderate HL probably due to primarily outer hair cell damage especially for frequencies above 4 kHz (LC2; Figure 1, upper right chart). This group was entitled “bilateral high frequency (HF) hearing loss.” Tinnitus patients with nearly normal audiogram (LC3; Figure 1, lower left chart) comprise about 20.6% of the total sample. Here, in rare cases (about 10% of this group), only frequencies above 4 kHz are involved with mild/moderate HL for both ears. A large proportion of patients with at least moderate HL in higher frequencies (2 kHz and above) for both ears can be observed in LC4. Twenty to thirty percent of this latent class were measured with thresholds over 50 dB above 4 kHz. Lower frequencies (below 500 Hz) are mostly not affected by HL. The proportion of this group is 13% of the total sample. The group was entitled “bilateral medium-high frequency HL.”

**Figure 2:**
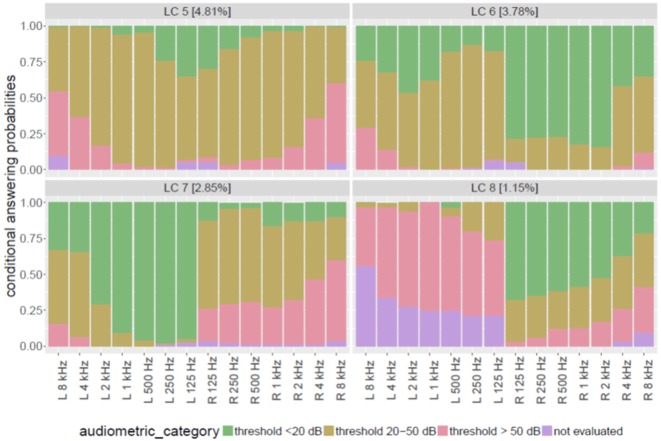
**Patterns of hearing loss with low prevalence in tinnitus patients**.

Figure 2 displays patterns of HL with much smaller proportion among tinnitus patients (all <5%). LC5 (upper left chart in Figure 2) was called “severe pantonal HL” and is characterized by high proportions of at least moderate HL at all measured frequencies. Almost half of the patients of this group have thresholds over 50 dB above 4 kHz. Both ears are concerned quite similarly.

By contrast, latent classes 6, 7, and 8 all display asymmetric patterns of HL. In LC6 (3.8% of total sample; upper right chart in Figure 2), most patients have normal hearing at the right ear below 4 kHz but severely impaired hearing at their left ear at all frequencies. Damage on the left ear is mostly a HL between 25 and 50 dB (“mild to moderate”). We therefore named this group “left-sided pantonal medium HL.”

It is noteworthy, that LC7 (2.9%, lower left chart of Figure 2) is not a symmetrical counterpart to LC6, though in this group mostly the right ear is affected by HL. But whereas LC6 members displayed mild to moderate HL at their affected left ear, members of LC7 have a broadband severe HL at their right ears. Already at 125 Hz, more than 20% of this group suffer from threshold elevations above 50 dB. This proportion is continuously increasing with increasing frequencies up to nearly 60% at 8 kHz. We therefore called this group of patients “right-sided pantonal severe HL.”

The smallest group of patients isolated by LCA can be depicted from the lower right chart of Figure 2 (LC8). In 1.2% of all patients, a pattern was observed with considerable proportion of normal hearing at the right ear in lower frequencies (e.g., 66% at 125 kHz), continuously decreasing to 20% at 8 kHz. But at patients’ left ears, nearly nobody was measured with normal hearing at any frequency: mostly, a severe HL (thresholds >50 dB) could be observed across all frequencies, or the left ear was not assessed (especially at higher frequencies). As practically, all patients had a full examination of the right ear, it can be assumed that missing values for left ears in this group do not mean “unknown” degree of HL, but represent a physician’s or audiologist’s decision to stop the assessment, because complete deafness of the left ear impede a full measurement of all frequencies at the left side. This group therefore was named “left-sided pantonal severe HL.”

Latent class 1 (lacking audiometry) during all statistical comparisons of clinical and demographic parameters yielded results very similar to the total average or proportions of the total sample (see Table 2). Therefore, no hints are present that the lack of an audiometric test was related to a certain subgroup of tinnitus patients. The relative size of the remaining seven HL-patterns thus could be adjusted to the proportions given in Table 2. These reflect the expected latent class sizes in our patient sample, if all patients had received threshold measurements.

Mean age of patients (see Table 2) was 51.7 years (SD 12.9) with the youngest patients presenting in LC3 (normal hearing) and the oldest patients in LC5 (severe pantonal HL). Female patients (total: 32.4%, see Table 2) were under-represented in LC2 (bilateral HF HL) and LC4 (bilateral medium-HF HL), and at largest over-represented in LC5 (severe pantonal HL) and LC6 (left-sided pantonal medium HL).

Age at onset of tinnitus was on average 43.2 years (SD 13.9; Table 2). Thus, there was a mean delay of nearly 9 years until presenting at the Regensburg Tinnitus Center. Patients with more or less normal hearing (LC3) had virtually no delay in help seeking. The small difference between age at onset as compared to biographical mean age (41.7 vs. 41.9 years) is probably confounded by missing values about the onset. Patients with bilateral medium-to-severe HF HL (LC4) and with severe pantonal HL (LC5) reported the latest onset of tinnitus.

Symptom load as measured by the Tinnitus Questionnaire (TQ, 20) was highest in LC5 (severe pantonal HL), and lowest in LC3 (normal hearing). Signs of depression as measured by BDI were also most prominent in LC5, and quite lenient in all other patient groups. For the TBF Questionnaire, a short form of the Tinnitus Handicap Inventory, mean scores 2 points above the total average were found for LC5 and LC8 (left-sided pantonal severe HL). Whereas patients’ self-ratings of tinnitus severity on the clinical global impression scale of tinnitus (CGI self-rating) revealed no significant differences between the different latent classes, the answers to the question “How much of a problem is your tinnitus?” differed significantly. But this effect was very small with membership in latent classes of HL explaining only less than 3% of the variance of patients’ ratings.

Characteristics of tinnitus manifestation over groups of HL are also given in Table 2. The different latent classes differed in most but not all characteristics (see Table 2). Whether or not tinnitus started abruptly, differed most between LC4 (43% abrupt start) and LC5 (40%) on the one side, and over 60% for latent classes 6 (left-sided pantonal medium HL) and 7 (right-sided pantonal severe HL). A non-pulsatile tinnitus was in total reported by 79.6% of all patients. Only in LC7 (right-sided pantonal severe HL) this proportion was considerably smaller (66%).

Handedness of patients could not be shown to covary with patterns of HL (and therefore was also not connected to side of HL). On which side patients experienced their tinnitus (exclusively on one side or with dominance of one side vs. bilateral symptoms) was clearly connected to the asymetrical patterns of HL. Members of LC6 (left-sided pantonal HL) as well as members of LC8 (left-sided pantonal severe HL) reported tinnitus more often for their left side. Accordingly, patients in LC7 (right-sided pantonal severe HL) clearly reported more often to experience their tinnitus on the right side (48% as compared to 12.3% in the total sample).

For LC8 (left-sided pantonal severe HL), their tinnitus was more frequently experienced as constant over time. If patients suffered from HF HL (LC2) or had quite normal audiometric results (LC3), they tended more often to describe their tinnitus as a tonal event. Patients with severe pantonal HL (LC5) by contrast had much higher proportions of tinnitus experienced as a kind of noise. The tinnitus pitch did not differ too much across the LCs except a smaller proportion of very high pitch (19% as compared to 28% for the whole sample) in LC7 (right-sided pantonal severe HL), and more prominence of low pitch tinnitus in LC3 (normal hearing) and LC6 (left-sided pantonal medium HL) with 4.4 and 6.3% (total sample: 2.5%).

## Discussion

In the present study, we performed a data-driven analysis of a large sample to identify clinically meaningful subtypes of HL patterns among tinnitus patients. Information about hearing function was derived from standard pure-tone audiograms, which were all performed in the context of clinical routine assessment according to standard procedures in one clinical center.

A potential limitation of our study is the limited sensitivity of the standard audiogram. The database only contained audiogram data from air conduction measurements, which made it impossible to differentiate cochlear from conductive HL. Moreover measurement of otoacoustic emissions might provide a more exact measurement of outer hair cell function than audiometric thresholds and it also remains to be determined whether the cut-offs for categorization of HL used in this study (<20 dB HL, 25–50 dB HL, >50 dB HL) are most appropriate. Finally, cochlear damage in the HF range above 8 kHz (21), dead regions between tested frequencies (22) and synaptopathy of high-threshold fibers (23) can all be relevant for tinnitus development but are not detected by the standard audiogram.

Presumably the different forms of “hidden hearing loss” are particularly relevant in LC3, the group with normal audiogram. However, in spite of the fact that patient categorization was only based on the audiogram which represents a rather rough information of cochlear function, our analysis revealed several relevant findings.

First, the classification algorithm identified eight distinct latent classes with an excellent separation. This means that all patients could be almost unambiguously allocated to a given class.

Second, the HL patterns of the different classes reflected typical clinical patterns of HL: bilateral normal hearing (LC3), bilateral HF HL (LC2), bilateral medium to HF HL (LC4), bilateral pantonal HL (LC5), medium (LC6), severe (LC8) pantonal HL left, and medium-to-severe pantonal HL right (LC7).

Third, patients of the various latent classes differed in most demographic and clinical tinnitus characteristics indicating the clinical relevance of our categorization and confirming HL as a relevant criterion for profiling of tinnitus patients.

As expected from the fact that bilateral HL frequently develops over the life span, patients with normal audiogram (LC3) were younger, whereas patients with pantonal pronounced HL (LC5) were older than average. This fits with the pattern of HL in LC5 which is typical for age-related HL. Another expected finding was that tinnitus laterality was related to the side of unilateral HL (LC6, LC7, LC8). Patients with unilateral HL (LC6, LC7) reported also more frequently abrupt tinnitus onset, which fits with sudden HL as a frequent cause of unilateral hearing impairment. By contrast, bilateral hearing impairment (LC4, LC5), which is typically developing slowly over time, was more often related to gradual onset.

Patients with HL exclusively in the HF range (LC2) and patients with normal audiogram (LC3), who frequently have hearing impairment in the extended HF range (21) had more often tonal tinnitus, which fits with their circumscribed hearing impairment. Accordingly, noise-like tinnitus was more frequently observed in patients with pantonal hearing impairment (LC5). Patients with pantonal impairment (LC5) were also characterized by higher tinnitus severity and more depressive symptoms, as reflected by increased scores in TQ, TBF-12, and BDI. This finding is in line with earlier investigations demonstrating that the degree of HL is associated with tinnitus severity (24).

In summary, our findings revealed distinct subtypes that reflect prototypic forms of HL and that differ in several relevant clinical characteristics. Further research should aim at further refinement by taking into account more detailed audiological informations. Finally, other possible approaches for cluster analyses should be tested and compared. One possible approach would be to weight differences between adjacent frequencies, as they might be particularly relevant for tinnitus generation (25). Such an approach may help to better identify relevant characteristics of hearing function (for example, the 4 kHz dip as a typical pattern of HL among tinnitus patients), which may remain insufficiently represented by a classification algorithm in which all tested frequencies are weighted equally.

## Author Contributions

BL, ML, HF, and UF designed the study; ML, MS, VV, TS, and SS were responsible for data management; HF and UF performed the statistical analysis; BL, ML, WS, and ML interpreted the results; BL and UF drafted the manuscript; all the authors contributed to the manuscript and approved first final version.

## Conflict of Interest Statement

The authors declare that the research was conducted in the absence of any commercial or financial relationships that could be construed as a potential conflict of interest.
