# Supplementary material for: Different Patterns of Hearing Loss among Tinnitus Patients: A Latent Class Analysis of a Large Sample
Source: Front Neurol. 2017 Feb 20;8:46. doi: 10.3389/fneur.2017.00046 (PMC5316929; doi:10.3389/fneur.2017.00046)
Supplement: Supplementary file 1 [file Data_Sheet_1.DOCX]

**Supplementary material: Calculation of latent classes**

Given the parameters of the LCA, one can calculate the probability of a certain pattern x of hearing loss to belong to latent class g by using the following formula.

$$P\left( g | \vec{x} \right)= \frac{\pi_{g}\prod_{i=1}^{I} \prod_{s=1}^{c_{i}} \pi_{isg}^{x_{is}}}{\sum_{j=1}^{G} \pi_{j} \prod_{i=1}^{I} \prod_{s=1}^{c_{i}} \pi_{isj}^{x_{is}}}$$

with

$\pi_{g}$ prior probability for class g (class size), g = 1, .., G

$\pi_{isg}$ probability for response in category s to item i in class g,
 i = 1, .. , I and s = 1, .. ,c_i_

$x_{is}$ indicator for response in category s to item i

$\vec{x}$ response pattern over all items

| **Side** | **Frequency** | **Category of Hearing Loss** $\pi_{isg}$ | | | | **LC number** | **Size LC** $\pi_{g}$ |
| --- | --- | --- | --- | --- | --- | --- | --- |
|  |  | **normal** | **external** | **internal** | **not evaluated** |  |  |
| Left | 125 Hz | 0 | 0 | 0 | 1 | 1 | 0.32168 |
| Right | 125 Hz | 0 | 0 | 0 | 1 | 1 |  |
| Left | 250 Hz | 0.001 | 0 | 0 | 0.999 | 1 |  |
| Right | 250 Hz | 0 | 0 | 0 | 1 | 1 |  |
| Left | 500 Hz | 0 | 0.001 | 0 | 0.999 | 1 |  |
| Right | 500 Hz | 0 | 0 | 0 | 1 | 1 |  |
| Left | 1 kHz | 0 | 0 | 0.001 | 0.999 | 1 |  |
| Right | 1 kHz | 0 | 0 | 0 | 1 | 1 |  |
| Left | 2 kHz | 0 | 0 | 0.001 | 0.999 | 1 |  |
| Right | 2 kHz | 0 | 0 | 0 | 1 | 1 |  |
| Left | 4 kHz | 0 | 0 | 0 | 1 | 1 |  |
| Right | 4 kHz | 0 | 0 | 0 | 1 | 1 |  |
| Left | 8 kHz | 0 | 0 | 0 | 1 | 1 |  |
| Right | 8 kHz | 0 | 0 | 0 | 1 | 1 |  |
| Left | 125 Hz | 0.959 | 0.001 | 0 | 0.04 | 2 | 0.2158 |
| Right | 125 Hz | 0.94 | 0.019 | 0 | 0.04 | 2 |  |
| Left | 250 Hz | 0.995 | 0.003 | 0 | 0.002 | 2 |  |
| Right | 250 Hz | 0.986 | 0.014 | 0 | 0 | 2 |  |
| Left | 500 Hz | 0.999 | 0.001 | 0 | 0 | 2 |  |
| Right | 500 Hz | 0.991 | 0.009 | 0 | 0 | 2 |  |
| Left | 1 kHz | 0.998 | 0.002 | 0 | 0 | 2 |  |
| Right | 1 kHz | 0.996 | 0.004 | 0 | 0 | 2 |  |
| Left | 2 kHz | 0.909 | 0.09 | 0 | 0.002 | 2 |  |
| Right | 2 kHz | 0.93 | 0.07 | 0 | 0 | 2 |  |
| Left | 4 kHz | 0.213 | 0.769 | 0.015 | 0.003 | 2 |  |
| Right | 4 kHz | 0.318 | 0.671 | 0.011 | 0 | 2 |  |
| Left | 8 kHz | 0.122 | 0.794 | 0.081 | 0.004 | 2 |  |
| Right | 8 kHz | 0.135 | 0.78 | 0.082 | 0.003 | 2 |  |
| Left | 125 Hz | 0.959 | 0.005 | 0 | 0.036 | 3 | 0.2061 |
| Right | 125 Hz | 0.962 | 0.002 | 0 | 0.036 | 3 |  |
| Left | 250 Hz | 1 | 0 | 0 | 0 | 3 |  |
| Right | 250 Hz | 1 | 0 | 0 | 0 | 3 |  |
| Left | 500 Hz | 1 | 0 | 0 | 0 | 3 |  |
| Right | 500 Hz | 0.995 | 0.005 | 0 | 0 | 3 |  |
| Left | 1 kHz | 0.996 | 0.004 | 0 | 0 | 3 |  |
| Right | 1 kHz | 0.985 | 0.015 | 0 | 0 | 3 |  |
| Left | 2 kHz | 0.98 | 0.018 | 0 | 0.002 | 3 |  |
| Right | 2 kHz | 0.989 | 0.009 | 0 | 0.002 | 3 |  |
| Left | 4 kHz | 0.875 | 0.125 | 0 | 0 | 3 |  |
| Right | 4 kHz | 0.921 | 0.079 | 0 | 0 | 3 |  |
| Left | 8 kHz | 0.871 | 0.121 | 0.001 | 0.007 | 3 |  |
| Right | 8 kHz | 0.889 | 0.103 | 0 | 0.008 | 3 |  |
| Left | 125 Hz | 0.935 | 0.029 | 0 | 0.036 | 4 | 0.1305 |
| Right | 125 Hz | 0.941 | 0.023 | 0 | 0.035 | 4 |  |
| Left | 250 Hz | 0.994 | 0.006 | 0 | 0 | 4 |  |
| Right | 250 Hz | 0.995 | 0.005 | 0 | 0 | 4 |  |
| Left | 500 Hz | 0.869 | 0.131 | 0 | 0 | 4 |  |
| Right | 500 Hz | 0.883 | 0.117 | 0 | 0 | 4 |  |
| Left | 1 kHz | 0.649 | 0.344 | 0.007 | 0 | 4 |  |
| Right | 1 kHz | 0.675 | 0.325 | 0 | 0 | 4 |  |
| Left | 2 kHz | 0.17 | 0.8 | 0.027 | 0.003 | 4 |  |
| Right | 2 kHz | 0.226 | 0.742 | 0.032 | 0 | 4 |  |
| Left | 4 kHz | 0.057 | 0.732 | 0.209 | 0.003 | 4 |  |
| Right | 4 kHz | 0.072 | 0.714 | 0.214 | 0 | 4 |  |
| Left | 8 kHz | 0.073 | 0.578 | 0.327 | 0.023 | 4 |  |
| Right | 8 kHz | 0.088 | 0.531 | 0.371 | 0.011 | 4 |  |
| Left | 125 Hz | 0.349 | 0.585 | 0.015 | 0.052 | 5 | 0.0481 |
| Right | 125 Hz | 0.296 | 0.615 | 0.037 | 0.052 | 5 |  |
| Left | 250 Hz | 0.24 | 0.746 | 0.015 | 0 | 5 |  |
| Right | 250 Hz | 0.159 | 0.804 | 0.037 | 0 | 5 |  |
| Left | 500 Hz | 0.043 | 0.938 | 0.019 | 0 | 5 |  |
| Right | 500 Hz | 0.078 | 0.856 | 0.067 | 0 | 5 |  |
| Left | 1 kHz | 0.058 | 0.898 | 0.044 | 0 | 5 |  |
| Right | 1 kHz | 0.033 | 0.885 | 0.082 | 0 | 5 |  |
| Left | 2 kHz | 0.008 | 0.821 | 0.17 | 0 | 5 |  |
| Right | 2 kHz | 0.033 | 0.81 | 0.158 | 0 | 5 |  |
| Left | 4 kHz | 0.002 | 0.63 | 0.367 | 0.001 | 5 |  |
| Right | 4 kHz | 0.002 | 0.638 | 0.359 | 0.001 | 5 |  |
| Left | 8 kHz | 0.006 | 0.446 | 0.453 | 0.095 | 5 |  |
| Right | 8 kHz | 0.007 | 0.392 | 0.551 | 0.051 | 5 |  |
| Left | 125 Hz | 0.171 | 0.755 | 0.009 | 0.065 | 6 | 0.0378 |
| Right | 125 Hz | 0.782 | 0.162 | 0 | 0.055 | 6 |  |
| Left | 250 Hz | 0.127 | 0.854 | 0.009 | 0.009 | 6 |  |
| Right | 250 Hz | 0.775 | 0.225 | 0 | 0 | 6 |  |
| Left | 500 Hz | 0.178 | 0.813 | 0.009 | 0 | 6 |  |
| Right | 500 Hz | 0.77 | 0.23 | 0 | 0 | 6 |  |
| Left | 1 kHz | 0.376 | 0.624 | 0 | 0 | 6 |  |
| Right | 1 kHz | 0.822 | 0.178 | 0 | 0 | 6 |  |
| Left | 2 kHz | 0.464 | 0.515 | 0.021 | 0 | 6 |  |
| Right | 2 kHz | 0.841 | 0.159 | 0 | 0 | 6 |  |
| Left | 4 kHz | 0.318 | 0.544 | 0.138 | 0 | 6 |  |
| Right | 4 kHz | 0.418 | 0.558 | 0.024 | 0 | 6 |  |
| Left | 8 kHz | 0.241 | 0.465 | 0.29 | 0.004 | 6 |  |
| Right | 8 kHz | 0.349 | 0.53 | 0.112 | 0.009 | 6 |  |
| Left | 125 Hz | 0.95 | 0.023 | 0 | 0.027 | 7 | 0.0285 |
| Right | 125 Hz | 0.124 | 0.615 | 0.222 | 0.039 | 7 |  |
| Left | 250 Hz | 0.975 | 0.013 | 0 | 0.012 | 7 |  |
| Right | 250 Hz | 0.041 | 0.662 | 0.272 | 0.024 | 7 |  |
| Left | 500 Hz | 0.959 | 0.041 | 0 | 0 | 7 |  |
| Right | 500 Hz | 0.038 | 0.653 | 0.296 | 0.012 | 7 |  |
| Left | 1 kHz | 0.905 | 0.095 | 0 | 0 | 7 |  |
| Right | 1 kHz | 0.161 | 0.57 | 0.257 | 0.012 | 7 |  |
| Left | 2 kHz | 0.706 | 0.294 | 0 | 0 | 7 |  |
| Right | 2 kHz | 0.125 | 0.55 | 0.312 | 0.012 | 7 |  |
| Left | 4 kHz | 0.343 | 0.593 | 0.064 | 0 | 7 |  |
| Right | 4 kHz | 0.126 | 0.408 | 0.454 | 0.012 | 7 |  |
| Left | 8 kHz | 0.326 | 0.518 | 0.156 | 0 | 7 |  |
| Right | 8 kHz | 0.101 | 0.297 | 0.565 | 0.037 | 7 |  |
| Left | 125 Hz | 0 | 0.262 | 0.523 | 0.215 | 8 | 0.0115 |
| Right | 125 Hz | 0.677 | 0.292 | 0.031 | 0 | 8 |  |
| Left | 250 Hz | 0 | 0.2 | 0.584 | 0.215 | 8 |  |
| Right | 250 Hz | 0.646 | 0.292 | 0.062 | 0 | 8 |  |
| Left | 500 Hz | 0.031 | 0.065 | 0.658 | 0.246 | 8 |  |
| Right | 500 Hz | 0.615 | 0.263 | 0.122 | 0 | 8 |  |
| Left | 1 kHz | 0 | 0 | 0.754 | 0.246 | 8 |  |
| Right | 1 kHz | 0.585 | 0.29 | 0.126 | 0 | 8 |  |
| Left | 2 kHz | 0 | 0.062 | 0.662 | 0.277 | 8 |  |
| Right | 2 kHz | 0.523 | 0.308 | 0.169 | 0 | 8 |  |
| Left | 4 kHz | 0 | 0.031 | 0.63 | 0.338 | 8 |  |
| Right | 4 kHz | 0.369 | 0.37 | 0.23 | 0.031 | 8 |  |
| Left | 8 kHz | 0 | 0.031 | 0.411 | 0.558 | 8 |  |
| Right | 8 kHz | 0.215 | 0.37 | 0.319 | 0.096 | 8 |  |
